# Vitamin D Insufficiency Is Associated with Lower Physical Function in Patients with Heart Failure and Diabetes

**DOI:** 10.1155/2014/320930

**Published:** 2014-08-25

**Authors:** M. R. Lopes, Paula A. B. Ribeiro, Priscila Ledur, Gabriela C. Souza, Nadine Clausell, Beatriz D. Schaan

**Affiliations:** ^1^Endocrinology Post-Graduate Program, Universidade Federal do Rio Grande do Sul, Porto Alegre, RS, Brazil; ^2^Cardiology Post-Graduate Program, Universidade Federal do Rio Grande do Sul, Porto Alegre, RS, Brazil; ^3^Hospital de Clínicas de Porto Alegre, Porto Alegre, RS, Brazil; ^4^Department of Internal Medicine, Faculty of Medicine, Universidade Federal do Rio Grande do Sul, Porto Alegre, RS, Brazil; ^5^Cardiology Division, Hospital de Clínicas de Porto Alegre, Porto Alegre, RS, Brazil; ^6^Endocrinology Division, Hospital de Clínicas de Porto Alegre, Serviço de Endocrinologia, Rua Ramiro Barcelos 2350, Prédio 12, 4° Andar, 90035-003 Porto Alegre, RS, Brazil

## Abstract

Vitamin D deficiency is frequent among patients with heart failure (HF) and diabetes, disorders associated with exercise intolerance and muscle weakness. This study aims to search for associations between vitamin D sufficiency and physical function indexes in patients with HF and diabetes. A cross-sectional study of 146 HF patients, 39.7% with diabetes, at a Brazilian tertiary outpatient clinic was performed. Patients underwent clinical evaluation, 6-minute walk test (6 MWT), handgrip strength, physical activity level (IPAQ), and biochemical evaluations including serum 25-hydroxyvitamin D. Classification was done according to vitamin D status (≥30 ng/dL, sufficient) and presence/absence of diabetes in vitamin sufficient, no diabetes (DS-C, *n* = 25), vitamin sufficient, diabetes (DS-DM, *n* = 18), vitamin deficient, no diabetes (DD-C, *n* = 63), and vitamin deficient, diabetes (DD-DM, *n* = 40). Patients age was 55.4 ± 8 yrs; 70.5% had vitamin D deficiency. Clinical characteristics were similar among groups. Total time expended in physical activity was similar among groups (*P* = 0.26). DS-C covered higher distances in the 6 MWT (392 ± 60 m) *versus* DD-DM (309 ± 116 m); *P* = 0.024. Handgrip strength was similar among groups but tended to lower levels in DD-DM (*P* = 0.074) even after being adjusted to physical activity (*P* = 0.069). Vitamin D deficiency can influence physical function in HF diabetic patients.

## 1. Introduction 

Congestive heart failure is a major cause of morbidity and mortality, with high costs to society [1]; its consequences are even worse when associated with diabetes mellitus. Heart failure incidence in patients with diabetes mellitus is much higher than in the general population [2] and is related to lower survival and lower responsiveness to treatments [3].

Patients with heart failure can develop a wasting process in which neurohormones and proinflammatory cytokines contribute to a catabolic process. Malabsorption from the gut as a result of bowel wall edema and decreased bowel perfusion also plays an important role, contributing to the progression of nutritional deficiency [4]. Importantly, the long-term prognosis is considerably worse once cardiac cachexia has been diagnosed [5].

Vitamin D deficiency has been associated with type 2 diabetes [6], hypertension [7], acute myocardial infarction [8], and heart failure [9], and its prevalence is rising around the world [10]. These are observational data, and association between vitamin D and those conditions can be confounded by factors such as general health status, exercise tolerance, obesity, and exposure to sunlight [11]. Vitamin D also plays a key role in muscle contraction [12] and has been related to muscle pain and weakness, fatigue, and performance, derangements that can be reversed by its oral supplementation [13]. Diabetes, especially when poorly controlled and of long-standing duration, can cause myopathy [14], which, if associated with vitamin D deficiency and heart failure, can determine major muscle impairment, with consequent low physical function and strength [15, 16]. These interactions could contribute to low levels of physical activity usually seen in individuals with diabetes [17] and heart failure [18].

There is no evidence from patients with heart failure and diabetes on the possible association of physical activity, physical performance, and low levels of vitamin D. Therefore, we aimed to investigate whether serum vitamin D and diabetes mellitus could be associated with physical function and physical activity behavior in patients with heart failure.

## 2. Material and Methods

### 2.1. Patients

This cross-sectional study enrolled 147 consecutive patients from the heart failure clinic of a tertiary, teaching general hospital. Inclusion criteria were age 30–65 years and a diagnosis of heart failure by the medical team. Exclusion criteria were pregnancy, chemotherapy, pacemakers or implantable cardioverter defibrillators, use of supplemental vitamins, chronic renal failure (glomerular filtration rate <30 mL/min), and HIV patients. This study was approved by the Institutional Ethics Committee (09-511).

### 2.2. Protocol

On the first day, patients were invited to participate in the study, the consent was applied, and they were instructed to come to the next visit 12 hours fasted. On the second day, patients underwent blood collection and completed a food recall record and physical activity questionnaire. Measurements of arterial pressure, ankle-brachial index, anthropometric measures, handgrip, and six-minute walk test (6 MWT) were performed. A subsample of patients who did not have any contraindication for a cardiopulmonary test was invited to participate in this last part of the protocol, in a third visit.

#### 2.2.1. Food Record

This was applied by a trained researcher (M.R.S.), estimating what was eaten in the day before the visit, evaluating macro- and micronutrients through the software Diet Win Professional 2008 (Porto Alegre, Brazil).

#### 2.2.2. Physical Activity Evaluation

To estimate time expended on physical activity we used the long version of the International Physical Activity Questionnaire (IPAQ) [19], which provides a physical activity evaluation in four different domains: working physical activity, housework physical activity, transport-related physical activity, and leisure-time physical activity. We also created a new parameter which was found to be of interest in this particular population of patients, which was the sum of questions about walking physical activity.

#### 2.2.3. Anthropometric Measurements

Weight (kg) was measured with subjects in light clothing and barefoot to the nearest 100 g with a digital scale (Toledo, model 2096PP, Sao Paulo, Brazil). Height (cm) was measured with barefoot participants positioned with heels, buttocks, back and head against the wall, and the head aligned in the Frankfort horizontal plane, to the nearest 0.1 cm using a stadiometer (Veeder-Root, Dyfed, UK). Body mass index [BMI = weight (kg)/height (m^2^)] was calculated.

Waist and hip circumferences were measured to the nearest 0.1 cm with a measuring tape. Waist circumference was measured at the narrowest point between the lower costal border and the iliac crest at the end of normal expiration, while hip circumference was measured at the maximum circumference of the buttocks in a horizontal plane.

Tricipital skinfolds were measured with a scientific adipometer (Cescorf, Porto Alegre, Brazil) with a scale of 0.1 mm. Skinfold thickness measurements were performed as per the International Standards for Anthropometric Assessment Manual. All measurements were performed on the right-hand side of the body, unless otherwise stated. Two measurements were taken and if the difference was greater than 7.5% a third measurement was performed. The final measurements were recorded to the nearest 0.1 mm and were reported as the average of two measurements or the median of three. To measure the arm circumference, the person was asked to stand with his/her arms relaxed at his/her side. The midpoint between the most superior and lateral point of the acromion border and the most proximal and lateral border of the head of the radius was determined, measuring at this point, at eye level, with constant tension applied to the tape. With the tape still around the midpoint of the arm, a mark was made on the most posterior point of the triceps (just below the measuring tape) area to assist in locating the triceps skinfold landmark. To measure the triceps skinfold thickness, the person was asked to stand relaxed with his/her right arm slightly pronated. The thumb and index finger were held parallel and used to grasp the skinfold, ensuring that the skin was rolled from side to side to remove any muscle. To measure skinfold thickness, the indicator on the callipers was zeroed. The callipers were placed at 90° to the skin, one centimeter distal to the marked skinfold site with the measurement taken after two seconds.

*Body composition* was evaluated using bioimpedanciometry (Inbody230 analyzer, Biospace, Seoul, Republic of Korea) following standard protocol [20]. The patients were instructed about previous food intake and clothes to wear on the day of the evaluation.

*Blood pressure* was evaluated after 15 minutes of rest, with an automatic sphygmomanometer (OMRON Comfort III Visomat Incoterm, Kyoto, Japan) and appropriate cuff to arm circumference; the mean of two measures was calculated.

#### 2.2.4. Ankle-Brachial Index (ABI)

Measurements were performed according to a standard protocol using Doppler (DV 610, Franca, Brazil). A mercury manometer and cuffs to measure brachial pressure and inferior limb were used. The ABI was defined as the ratio between the highest systolic blood pressure of the ankle (posterior tibial or* dorsalis pedis* arteries) and highest systolic pressure of the arm (right or left). The index was calculated for each leg as the ratio between the averages of two measures of pressure on each limb. The cutoff point for diagnosis of peripheral arterial disease (PAD) was ABI ≤ 0.90 or ≥1.40 [21, 22]. This index was used to exclude patients with severe peripheral vascular disease, which could impair physical function evaluation.

#### 2.2.5. Handgrip Strength

Handgrip was used as general muscle strength evaluation. To measure and calculate weakness, we used a hydraulic hand dynamometer-Jamar (Sammons Preston, Bolingbrook, IL, USA). Maximal force of three tests with both hands was evaluated. The strength index was a mean of best try of dominant hand and cut-point of weakness (32.2 Kgf), age and gender predicted [23].

#### 2.2.6. Submaximal Functional Capacity

Functional capacity was assessed using the 6 min walk test (6 MWT) [24]. Patients were instructed to cover the greatest distance possible at a self-selected walking speed, in a 20 m corridor during the 6 minutes. They were accompanied by a staff member and encouraged by constant verbal stimulation, walking as fast as possible. During the test the heart rate was monitored (Polar, Kempele, Finland) to obtain resting, mean, and maximal heart rate.

#### 2.2.7. Cardiopulmonary Exercise Testing

Eighty-three patients who did not have any contraindication to perform a maximal test underwent symptom-limited testing on a treadmill (IMBRAMED, Porto Alegre, Brazil) using a ramp protocol, until exhaustion, as previously described [25]. In short, breath-by-breath gas exchange was continuously analyzed (Metalyzer 3B, Cortex, Leipzig, Germany). Peak oxygen uptake (VO_2 peak_) was defined as the highest value achieved during the test for 20 s. Heart rate and a 12-lead electrocardiogram were also continuously recorded.

#### 2.2.8. Biochemical Parameters

Blood samples were obtained in fasting state to measure the following parameters using commercial kits: glycemia, total cholesterol, high-density lipoprotein cholesterol (HDL-c), triglycerides (automated enzymatic commercial kits; Roche, Mannheim, Germany), insulin (enzyme immunoassay commercial kits; Abbot-Murex, Park, IL, USA), and C-reactive protein (nephelometry, nephelometer BN100, Dade Behring Inc., Marburg, Germany). Low-density lipoprotein cholesterol (LDL-c) was calculated using the Friedewald formula.

Calcium, magnesium, and albumin were evaluated using colorimetric techniques in a Modular P system (Roche, Basel, Switzerland). Albumin was evaluated with colorimetric bromocresol green, prothrombin time with the turbidimetric method (Dade Behring, Marburg, Alemanha). Glomerular filtration rate (GFR) was calculated with MDRD equation (modification of diet in renal disease) [26]. Parathyroid hormone was measured with an enzyme-linked immunosorbent assay (ELISA) (Bioamérica, Inc., Irvine, CA, USA) and read with SpectraMax M2 (Molecular Devices, Orleans, USA). The optic density data were compared to standard curve and calculated with linear regression, expressed in picograms per milliliters. Serum 25-hydroxyvitamin D levels were determined by radioimmunoassay, based on an antibody with specificity for 25-OH-D (DiaSorin, Stillwater, MN). Low serum 25-OH-vitamin was defined as values lower than 30 ng/dL [27].

### 2.3. Statistical Analysis

All values were expressed as mean ± standard deviation for variables with normal distribution and median and interquartile (p25–p75) to nonparametric variables. The analyses compared patients separated into the following categories: no vitamin deficiency and no diabetes (DS-C, *n* = 25), no vitamin deficiency and diabetes (DS-DM, *n* = 18), vitamin deficiency and no diabetes (DD-C, *n* = 63), and vitamin deficiency and diabetes (DD-DM, *n* = 40). Moreover, analyses were also performed considering the subgroup of patients with left ventricular dysfunction (left ejection fraction lower than 45%, Simpson or Teichholz, as indicated). Analysis of variance with multiple comparisons and Bonferroni correction was performed for comparisons. Possible correlations among variables were performed using Pearson Chi-square test. For all analyses SPSS version 17.0. was used (SPSS, Chicago). The statistical significance adopted was *P* < 0.05.

## 3. Results

A total of 1755 patients were screened, 1608 of whom were excluded according to the exclusion criteria and 123 declined to participate. One hundred and forty-seven patients were included in the final analysis, aged 55.4 ± 8 years, 95 (65.1%) men and 117 (80.7%) Caucasians. According to their vitamin D status, 43 (29.5%) were classified as vitamin D sufficient and 103 (70.5%) as vitamin D deficient. Among the 146 patients, 58 had diabetes mellitus. One hundred and fifteen patients had left ventricular dysfunction, and their data is described separately.

Clinical and biochemical characteristics according to vitamin D status and presence or absence of diabetes are shown in Table 1. There was no difference considering most of the characteristics across groups, except for BMI, which was higher in the DD-DM* versus* DD-C group (*P* = 0.007), and waist circumference, that followed the same pattern (*P* = 0.031). Previous myocardial infarction, bypass surgery, or percutaneous coronary intervention was more prevalent in the DD-DM group. Lower levels of hemoglobin were observed in the same group when compared to the DS-C group (*P* = 0.007). There was no difference when patients with left ventricular dysfunction were analyzed.

Table 2 shows food intake and parameters obtained from the 24 h food records. The amount of macronutrient intake, such as calcium and vitamin D, was similar across the groups. However, DD-DM had less caloric intake when compared to DD-C (*P* = 0.032). There was no difference when patients with left ventricular dysfunction were analyzed (data not shown).

**Figure 1:**
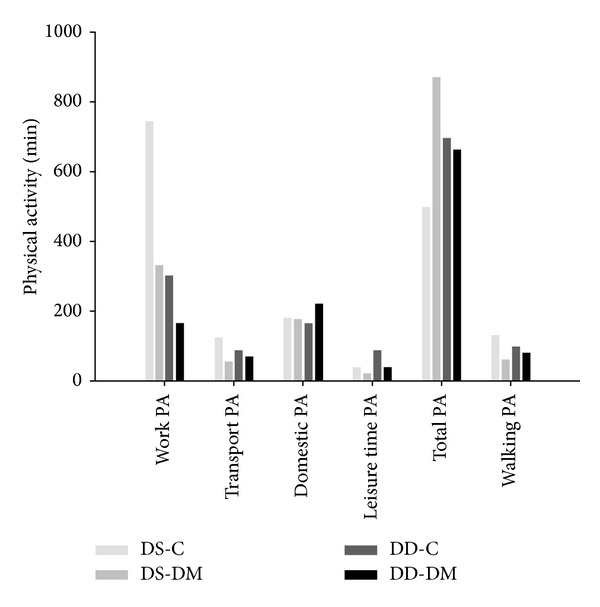
Physical activity (PA) expressed through the domains obtained from the IPAQ for the groups studied (no vitamin deficiency and no diabetes, DS-C; no vitamin deficiency and diabetes, DS-DM; vitamin deficiency and no diabetes, DD-C; and vitamin deficiency and diabetes, DD-DM).

Considering physical activity in minutes, total activity, activity during work, transport-related, in house, during leisure time, and walking were similar among groups (data not shown). The prevalence of physically active patients according to the WHO recommendation (>150 min/week) was 51.7%. However, the analyses of the individual physical activity domains disclose that the diabetes and vitamin D deficiency group reported less leisure time physical activity and walking (7.5% and 15%, resp.) than patients without diabetes and adequate vitamin D (12% and 24%, resp.), although these differences were not statistically significant (Figure 1). The proportion of patients that achieved the recommendation of physical activity was similar across the groups (*P* = 0.13). When analyzed in the different domains provided by the IPAQ, the numbers were work, 16.3% (*P* = 0.402); transport-related 15.6% (*P* = 0.560); house, 23.8% (*P* = 0.580); leisure, 9.5% (*P* = 0.830); and walking, 17.0% (*P* = 0.700).

On the other hand, the prevalence of no physical activity (score = zero) was 16% in the DS-C group, 33.3% in the DS-DM group, 27% in the DD-C group, and 15% in the DD-DM group. There was no difference across the groups, not even when analyzing patients with left ventricular dysfunction for all variables of physical activity.

Submaximal functional capacity evaluated by the 6 MWT showed that DD-DM had the worst performance compared to DS-C, considering a cutoff point of 300 m in the distance covered (Table 3). The same result was found when only patients with left ventricular dysfunction were analyzed. No differences were observed across the other groups. The maximal functional capacity, evaluated in a subsample of patients, is shown in Table 4. There was no difference in metabolic and ventilatory parameters across the groups. However, the VO_2_ peak had a tendency to significance when only patients with left ventricular dysfunction were analyzed (*P* = 0.059).

The handgrip strength was similar between groups. There was a tendency to less strength in the DD-DM group (25.4 ± 10 Kg) compared to others (DS-C: 33.5 ± 15 Kg, DS-DM: 29.8 ± 9 Kg, and DD-C: 30.4 ± 13 Kg, *P* = 0.074). However, when we analyzed only patients with left ventricular dysfunction, the results showed less strength in DD-DM* versus* DS-C (*P* = 0.027).

## 4. Discussion

The present study showed that heart failure patients with diabetes and vitamin D deficiency had lower physical function, albeit having similar clinical characteristics to those without vitamin D deficiency and no diabetes. Besides that, they had lower handgrip strength, which was progressively better through categories until the best clinical profile (patients without diabetes and sufficient vitamin D).

A high prevalence of vitamin D deficiency (70.5%) was observed in this sample. Interestingly, the association with diabetes did not show a higher prevalence of vitamin D deficiency, which indicates that causal factors are not related to diabetes but to heart failure syndrome and are in accordance with reports published in other diabetes subgroups [28]. However, this prevalence is lower than some studies that analyzed heart failure only, which implicated less exposure to sun [29] or lower vitamin D conversion to active metabolites due to skin characteristics [30, 31]. These results show the importance of taking ethnic characteristics into account and the place of data collection in this kind of analysis.

In our study, the four groups classified according to the presence of diabetes and vitamin D status were clinically similar, except for HbA1c and antidiabetics, and had a higher prevalence of previous cardiovascular events, as expected [32, 33]. The clinical similarity among groups was also comprehensible, as heart failure patients are managed in a reference ambulatory, following heart failure guidelines as strictly as possible. Interestingly, more previous cardiovascular disease was found in the group with vitamin D deficiency, irrespective of the presence of diabetes, and could result from increased risk associated with higher levels of inflammatory markers [32], endothelial dysfunction [34], and higher carotid intima-media thickness [35].

Low levels of hemoglobin and lower caloric intake in patients with diabetes and vitamin D deficiency suggest lack of adequate nutrition in this group, although they had a higher body mass index when compared to the others. This finding reinforces that obesity may be accompanied by undernutrition because of unhealthy choices [36, 37]. Moreover, as underreporting in dietary questionnaires is related to characteristics such as obesity, dieting, and psychological factors [38], this should also be considered.

Physical function impairment is expected in heart failure patients [39] because of symptoms related to the baseline disease, limb, and respiratory muscular weakness [40, 41] as well as impaired aerobic capacity [42]. Reduced aerobic capacity can be explained because of ventilatory dysfunction, muscle dysfunction, and sedentary behavior [18, 41]. Exercise intolerance is directly related to exercise dyspnea and early fatigue, which can be the major cause of low levels of physical activity in this population, and a vicious cycle clearly ensues. Vitamin D deficiency is positively correlated with worse aerobic capacity in heart failure [43, 44] and in healthy adults, an association that is stronger among those who reported the lowest level of physical activity [45]. We were able to show, for the first time, that vitamin D deficiency associated with diabetes results in higher impact on physical function than in each isolated condition, as evaluated by the 6 MWT.

Strength indexes have been used as prognostic and mortality indices in heart failure patients [41, 46]. In the general population, reduced handgrip strength is associated with aging [47], gender, and anthropometric measures [23]. Although our findings were not statistically significant in the whole sample, but different when only those with more severe disease were analyzed, the trend is consistent with the idea that patients with a worse clinical profile (with diabetes and vitamin D deficiency) have lower muscle strength than the other groups evaluated, independent of presenting other factors that contribute to this mechanism, such as low physical activity.

The prevalence of physically active subjects in this study was 51.7%, which is similar to data from healthy populations [48]. The analyses of the individual physical activity domains disclose that the diabetes and vitamin D deficiency group reported less leisure time physical activity and walking than patients without diabetes and adequate vitamin D. This is in accordance with findings in patients with chronic renal failure on dialysis: vitamin D is associated with lower levels of self-reported physical activity [49]. Considering functional capacity, muscle strength, and physical activity, it is hard to be sure which determines the other, and if they are merely associated. We speculate that physical function parameters can be associated with low levels of physical activity observed in these patients, such as muscle strength. This leads us to speculate that patients in a better physical condition can feel better when performing physical activities in everyday activities, maybe because they are less symptom-limited to exercise.

Our study has some limitations that should be pointed out. First, the possibility of reversal causality that is common in cross-sectional studies should be considered. Low levels of physical activities, as shown in our results, can predispose to not performing outside activities, and consequently less sun exposure and higher levels of vitamin D deficiency would be expected [50]. Second, underpowered analysis is a possibility, mostly in handgrip strength and cardiopulmonary testing variables, because sample size was not calculated taking these comparisons into account. And finally, physical function parameters for the whole sample analyzed were based on submaximal capacity, which is not the gold standard for this evaluation, even though it is well validated relative to ergospirometry [24].

## 5. Conclusion

In conclusion, patients with heart failure, diabetes, and vitamin D insufficiency show worse physical function and lower muscle strength compared to patients without diabetes and adequate vitamin D levels. However, even with no significant association, physical behavior has to be taken into account since it is directly related to physical function. We suggest that the treatment of these patients with pharmacological doses of vitamin D should be appropriately tested in a clinical trial to explore whether it can be of benefit to improve patients' outcome.

## Figures and Tables

**Table 1:** Clinical and biochemical characteristics according to vitamin D status and diabetes mellitus.

Characteristc	Vitamin D sufficient		Vitamin D insufficient		*P*
	(*n* = 43)		(*n* = 103)		
	No DM	DM	No DM	DM	
	*n* = 25	*n* = 18	*n* = 63	*n* = 40	
Male	17 (68.0)	13 (72.2)	39 (61.9)	26 (65.0)	0.855
Age (years)	54.4 ± 9	57.8 ± 6	53.1 ± 9	56.2 ± 6	0.051
Caucasian	19 (76.0)	17 (94.4)	49 (77.8)	32 (82.1)	0.106
Previous cardiovascular disease∗	9 (36)	7 (38.9)	38 (60.3)	25 (62.5)	0.050
LVEF (%)	39.1 ± 16	36.7 ± 11.9	31.5 ± 13.3	35.5 ± 14	0.101
Left ventricular dysfunction	18 (72)	14 (78)	55 (87)	28 (70)	0.166
Functional Class (NYHA)					
I	11 (47.8)	8 (57.1)	22 (36.7)	10 (33.3)	0.424
II	8 (34.8)	5 (35.7)	27 (45.0)	9 (30.0)	
III	4 (17.4)	1 (7.1)	9 (15.0)	9 (30.0)	
IV	0 (0.0)	0 (0.0)	2 (3.3)	2 (6.7)	
Drugs in use					
Metformin	—	12 (66.7)	—	23 (57.5)	<0.001
Sulfonyurea	—	5 (27.8)	—	9 (22.5)	<0.001
Insulin	—	4 (22.2)	—	12 (30.0)	<0.001
Diuretic	18 (72.0)	15 (83.3)	55 (87.3)	37 (92.5)	0.137
ACEi or ARA II	22 (88.0)	14 (77.8)	51 (81.0)	31 (77.5)	0.747
Aspirin	7 (28.0)	13 (72.2)	30 (47.6)	26 (65.0)	0.008
Mass (Kg)	82.1 ± 17	81.9 ± 13	75.3 ± 16	84.6 ± 20	0.992
BMI (Kg/m^2^)	28.7^AB^ ± 5	30.2^AB^ ± 5	27.7^A^ ± 5	31.5^B^ ± 6	0.007
Muscle mass (kg)	31.9 ± 7	30.4 ± 5	28.7 ± 7	32.1 ± 10	0.163
Fat mass (Kg)	25.4 ± 9	27.5 ± 10	24.2 ± 9	27.7 ± 12	0.339
Metabolic basal rate (Kilocalories)	1597 ± 265	1461 ± 400	1475 ± 248	1607 ± 342	0.078
Waist circumference (cm)	100.4^AB^ ± 11	105^AB^ ± 20	99.0^A^ ± 14	107.1^B^ ± 15	0.031
Arm circumference (cm)	33.2 ± 5	34.6 ± 4	32.3 ± 5	33.9 ± 4	0.154
Tricipital fold (mm)	18.6 ± 7	22.2 ± 9	20.0 ± 8	21.9 ± 9	0.375
Systolic arterial pressure (mmHg)	122.2 ± 22	115.9 ± 14	121.8 ± 23	130.4 ± 18.7	0.077
Diastolic arterial pressure (mmHg)	78.8 ± 12	76.6 ± 11	80.8 ± 19	81.9 ± 15	0.479
HbA1c (% and mmol/mol)	—	8.3 ± 3	—	7.7 ± 2	0.442
		(67 ± 2)		(61 ± 2)	
Total cholesterol (mg/dL)	188.5 ± 41	161.0 ± 28	178.8 ± 47	179.5 ± 69	0.440
HDL cholesterol (mg/dL)	41.7 ± 12	37.7 ± 9	38.1 ± 10	37.1 ± 8	0.382
Triglycerides (mg/dL)	149 (100–202)	163 (105–196)	153 (114–214)	138 (99–273)	0.524
Prothrombin time (s)	16.0 ± 8	15.3 ± 7	15.3 ± 7	16.0 ± 8	0.957
Calcium (mg/dL)∗∗	9.3 ± 1	9.3 ± 1	9.4 ± 1	9.5 ± 1	0.662
Magnesium (mg/dL)	2.1 ± 0	1.9 ± 0	2.1 ± 0	2.0 ± 0	0.060
PTH (pg/mL)	63.6 ± 20	62.7 ± 34	77.6 ± 38	78.3 ± 42	0.186
Hemoglobin (g/dL)	14.2^B^ ± 1	13.3^AB^ ± 2	13.7^AB^ ± 2	12.8^A^ ± 2	0.007
GFR (mL/min/m^2^)∗∗∗	95.4 ± 37	88.0 ± 35	86.7 ± 27	90.9 ± 40	0.739
CRP (mg/dL)	3.8 (1.7–6.9)	5.3 (2.1–8.8)	2.9 (1.1–6.7)	3.5 (1.0–10.6)	0.383

Mean ± standard deviation, *n* (%) or median (P25–P75), as needed. Different letters indicate statistically significant differences between groups (ANOVA with Tukey *post-hoc*). DM: diabetes mellitus; LVEF: left vetricular ejection fraction; GFR: glomerular fraction rate. ACEi: angiotensin converting enzyme inhibitors. ARA II: angiotensin II receptor blockers. Mass, body mass index (BMI), muscle mass, fat mass and basal metabolic rate were obtained using bioimpedanciometry. HbA1c (glycated hemoglobin), is expressed in NGSP units (%) and in IFCC units (mmol/mol). PTH: parathyroid hormone. ∗Previous cardiovascular disease included myocardial infarction, coronary artery bypass graft surgery and percutaneous coronary revascularization. ∗∗Corrected for albumin. ∗∗∗GFR was calculated with the MDRD equation.

**Table 2:** Food intake and caloric consumption according to vitamin D and diabetes mellitus.

Characteristic	Vitamin D sufficient		Vitamin D insufficient		*P*
	(*n* = 43)		(*n* = 103)		
	No DM	DM	No DM	DM	
	*n* = 25	*n* = 18	*n* = 63	*n* = 40	
Total caloric intake (Kcal)	1702^AB^ (1210–2461)	1455^AB^ (1250–1881)	1483^B^ (1133–2117)	1312^A^ (989–1572)	0.032
Carbohydrates (%)	48.3 (42–57)	50.7 (48–54)	55.3 (46–63)	56.5 (48–62)	0.158
Proteins (%)	17.9 (14–24)	16.8 (15–21)	18.4 (15–21)	19.5 (16–22)	0.603
Fat (%)	31.2 (25–48)	31.4 (28–35)	30.7 (22–39)	25.4 (20–37)	0.066
Calcium (mg/dL)	539 (285–679)	583 (289–741)	434 (237–688)	474 (286–760)	0.860
Vitamin D (mcg)	1.9 (1–3)	3.6 (2–7)	2.0 (0–4)	2.1 (1–4)	0.250

Median (P25–P75), as needed. Different letters indicate statistically significant differences between groups (Kruskall-Wallis). DM: diabetes mellitus.

**Table 3:** Six minutes walking test-submaximal physical function according to vitamin D and diabetes mellitus.

Characteristic	Vitamin D sufficient		Vitamin D insufficient		*P*
	(*n* = 43)		(*n* = 103)		
	No DM	DM	No DM	DM	
	*n* = 25	*n* = 18	*n* = 63	*n* = 40	
Distance covered (m)	398 (354–437)	344 (330–461)	375 (320–440)	325 (271–371)∗	0.014
≥300 m	24 (96.0)	14 (82.4)	51 (81.0)	26 (65.0)∗	0.024
Final HR (bpm)	101.6 ± 24	94.2 ± 32	97.6 ± 24	99.7 ± 32	0.835
Recovery HR (bpm)	86.4 ± 21	85.6 ± 18	82.8 ± 22	86.1 ± 20	0.825
Referred a high rate of perceived exertion^#^	16 (64.0)	14 (82.4)	39 (62.9)	21 (52.5)	0.204

Mean ± standard-deviation, *n* (%), or median (P25–P75), as needed. ANOVA and chi-square statistics. ∗Different from No DM, vitamin sufficient. DM: diabetes mellitus; HR: heart rate.

^#^Rate of perceived exertion was measured with the Borg Scale (6–20).

**Table 4:** Cardiopulmonary exercise test-metabolic and ventilatory characteristics according to vitamin D and diabetes mellitus.

Characteristic	Vitamin D sufficient		Vitamin D insufficient		*P*
	(*n* = 23)		(*n* = 60)		
	No DM	DM	No DM	DM	
	*n* = 14	*n* = 9	*n* = 38	*n* = 22	
VO^2^ peak (mL/kg/min)	20.0 ± 5	15.6 ± 3	18 ± 5	19.2 ± 5	0.134
≤14 mL mL/kg/min	1 (7.1)	3 (33.3)	8 (21.1)	2 (9.1)	
>14 mL mL/kg/min	13 (92.9)	6 (66.7)	30 (78.9)	20 (90.9)	0.250
VE/VCO^2^slope	35.8 ± 11	41.5 ± 5	37.3 ± 11	36.7 ± 9	0.636
>34	6 (42.9)	8 (100.0)	22 (62.9)	13 (65.0)	
≤34	8 (57.1)	0 (0.0)	13 (37.1)	7 (35.0)	0.066
Weber Class					
I	5 (35.7)	2 (22.2)	13 (34.2)	8 (36.4)	
II	8 (57.1)	1 (11.1)	16 (42.1)	9 (40.9)	
III	1 (7.1)	6 (66.7)	7 (18.4)	3 (13.6)	
IV	0 (0.0)	0 (0.0)	2 (5.3)	2 (9.1)	0.059
HR peak (bpm)	139.8 ± 31	141.9 ± 27	137.4 ± 24	134.9 ± 28	0.909
Systolic arterial pressure (mmHg)	146 ± 35	140.4 ± 22	137.1 ± 32	148.7 ± 25	0.516
Diastolic arterial pressure (mmHg)	74.5 ± 16	73.5 ± 9	71.6 ± 15	74.1 ± 14	0.893
O^2^ pulse (mL*·*btm)	11.61 ± 4	9.2 ± 2	10.9 ± 4	19.0 ± 30	0.236

Mean ± standard deviation, or *n* (%), as needed. ANOVA and Chi-square statistics. DM: diabetes mellitus. VO^2^ peak: peak oxigen consumption. Weber class: I: >20; II: 16–20; III: 10–16; IV: <10.
